# Airflow limitation in a collapsible model of the human pharynx: physical mechanisms studied with fluid‐structure interaction simulations and experiments

**DOI:** 10.14814/phy2.14099

**Published:** 2019-05-22

**Authors:** Trung B. Le, Masoud G. Moghaddam, B. Tucker Woodson, Guilherme J. M. Garcia

**Affiliations:** ^1^ Department of Biomedical Engineering Marquette University & The Medical College of Wisconsin Milwaukee Wisconsin; ^2^ Department of Otolaryngology and Communication Sciences Medical College of Wisconsin Milwaukee Wisconsin; ^3^ Present address: Department of Civil and Environmental Engineering North Dakota State University Fargo North Dakota

**Keywords:** Airflow limitation, fluid‐structure interaction simulations and experiments, obstructive sleep apnea, Starling Resistor biomechanical model of airway collapse, wave‐speed flow limitation theory

## Abstract

The classical Starling Resistor model has been the paradigm of airway collapse in obstructive sleep apnea (OSA) for the last 30 years. Its theoretical framework is grounded on the wave‐speed flow limitation (WSFL) theory. Recent observations of negative effort dependence in OSA patients violate the predictions of the WSFL theory. Fluid‐structure interaction (FSI) simulations are emerging as a technique to quantify how the biomechanical properties of the upper airway determine the shape of the pressure‐flow curve. This study aimed to test two predictions of the WSFL theory, namely (1) the pressure profile upstream from the choke point becomes independent of downstream pressure during flow limitation and (2) the maximum flowrate in a collapsible tube is $V_{I\max}=A^{3/2}{\left(\rho dA/dP\right)}^{-1/2}$, where *ρ* is air density and *A* and *P* are the cross‐sectional area and pressure at the choke point respectively. FSI simulations were performed in a model of the human upper airway with a collapsible pharynx whose wall thickness varied from 2 to 8 mm and modulus of elasticity ranged from 2 to 30 kPa. Experimental measurements in an airway replica with a silicone pharynx validated the numerical methods. Good agreement was found between our FSI simulations and the WSFL theory. Other key findings include: (1) the pressure‐flow curve is independent of breathing effort (downstream pressure vs. time profile); (2) the shape of the pressure‐flow curve reflects the airway biomechanical properties, so that $V_{I\max}$ is a surrogate measure of pharyngeal compliance.

## Introduction

**Figure 1:**
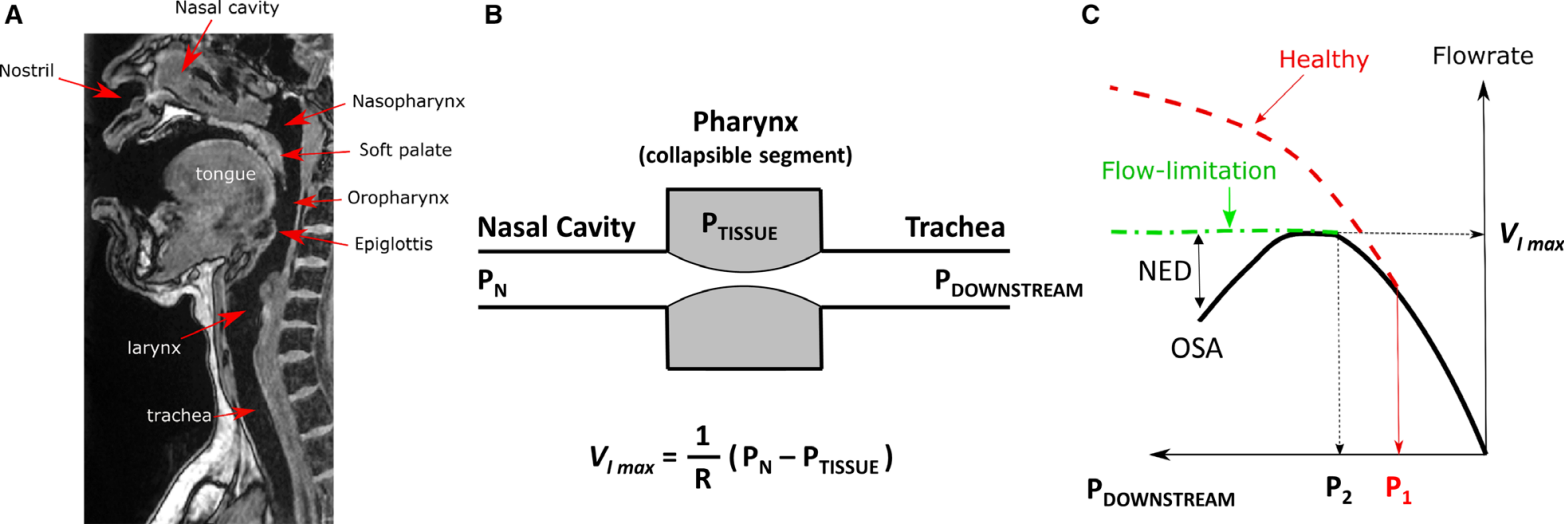
(A) Magnetic Resonance Imaging data of a healthy subject used to create the upper airway model. The soft palate is the most frequent site of airway collapse in patients with obstructive sleep apnea (OSA). (B) Diagram of the classical Starling Resistor model. (C) Airflow limitation occurs when the inspiratory flowrate plateaus at $V_{I\max}$ despite further reductions in downstream pressure. Some OSA patients even display negative effort dependence (NED). The pressure‐flow curve of OSA patients starts to deviate from the healthy profile at the pressure P_1_, whose magnitude is smaller than the pressure P_2_ at which flow limitation occurs.

Obstructive sleep apnea (OSA) is a disease characterized by recurrent episodes of airflow limitation during sleep caused by airway collapse at the pharynx (Fig. 1) (Gold and Schwartz 1996; Dempsey et al. 2010). Untreated OSA has many negative health consequences that reduce quality of life and decrease life expectancy (Young et al. 2008). Today, treatment is not successful for many patients. This problem persists because there is no reliable method to prospectively predict which patients will respond favorably to a given treatment modality, such as continuous positive airway pressure (CPAP), oral appliances, or surgery. Research suggests that classification of patients into different anatomical phenotypes based on the site of airway collapse can improve treatment efficacy (Vanderveken 2013; Vanderveken et al. 2013; Strollo et al. 2014). One method to classify anatomical phenotypes is by visualization of the dynamics of airway collapse during drug induced sedated endoscopy, but this method has several limitations, such as its high cost and significant level of expertise required to consistently identify the flow‐limiting site (Vanderveken 2018). Recently, it was proposed that relevant clinical information, including the site of airway collapse, can be extracted from the flow shapes recorded during polysomnography (Genta et al. 2017). This could provide a low cost and widely accessible method for evaluation of the biomechanical properties of the upper airway. However, the relationship between the biomechanical properties of the upper airway and the flow shapes recorded during polysomnography remains poorly understood (Aittokallio et al. 2001; Pamidi et al. 2017).

The precise mechanism of airway collapse in OSA is not completely understood. During the last 30 years, the classical Starling Resistor model has been the paradigm to explain the mechanism of airway collapse in OSA (Smith et al. 1988; Schwartz and Smith 2013). In a Starling Resistor (Fig. 1B), a collapsible tube (the pharynx) is mounted between a rigid upstream segment (the nasal cavity) and a rigid downstream segment (the trachea). The collapsible tube is enclosed by a sealed box where the external air pressure (tissue pressure) can be controlled. The tube collapses when air pressure in the tube lumen becomes less than the external pressure. The classical Starling Resistor model is based on the assumption that in flow‐limited breaths the peak inspiratory flow ($V_{I\max}$) is independent of the downstream pressure and determined by the tissue pressure surrounding the pharynx, namely (Smith et al. 1988; Schwartz and Smith 2013)

$$V_{I\max}=\frac{1}{R}\left(P_{N}-P_{\mathrm{tissue}}\right),$$

where *P*_*N*_ is the upstream pressure at the nostrils, *P*_tissue_ is the tissue pressure surrounding the pharynx, and R is the upstream resistance. The classical Starling Resistor model is supported by the experimental observation that $V_{I\max}$ increases linearly with CPAP pressure (*P*_*N*_) in OSA patients (Smith et al. 1988; Gold and Schwartz 1996). The theoretical framework of the classical Starling Resistor model is the wave‐speed flow limitation theory, which asserts that the pressure profile upstream from the choke point becomes independent of the downstream pressure after flow limitation starts (Dawson and Elliott 1977; Elliott and Dawson 1977).

The validity of the classical Starling Resistor model was recently questioned by Wellman and collaborators based on the observation of negative effort dependence in many OSA patients (Fig. 1C) (Butler et al. 2013; Owens et al. 2014; Wellman et al. 2014). Negative effort dependence occurs when inspiratory flow decreases (rather than increase) as the downstream pressure becomes more negative. To date, a complete theory to explain negative effort dependence in OSA patients is lacking (Butler et al. 2013). A better understanding of how the biomechanical properties of the upper airway determine the shape of the pressure‐flow curve may allow the future development of tools to classify anatomical phenotypes based on flow shapes recorded during polysomnography (Aittokallio et al. 2001; Azarbarzin et al. 2017; Genta et al. 2017; Pamidi et al. 2017). In addition, a better understanding of the biomechanics of upper airway collapse may allow the development of novel surgical techniques that are more effective at preventing airway collapse.

Fluid‐structure interaction (FSI) simulations is a computational method to quantify the coupling between the motions of a fluid and a structure, including structural deformation in response to forces imposed by the fluid. In recent years, several FSI studies have investigated airway collapse in OSA patients (Pirnar et al. 2015; Subramaniam et al. 2017; Chang et al. 2018; Liu et al. 2018). However, most FSI studies were limited to relatively small deformations and thus they did not quantify airflow limitation. In addition, with few exceptions (Chouly et al. 2008; Zhao et al. 2013), most previous studies lacked experimental validation of the FSI methodology.

The objectives of this study were to (1) develop and validate an FSI methodology to simulate upper airway collapse, (2) quantify how the peak flowrate $V_{I\max}$ in a collapsible model of the upper airway depends on wall thickness and modulus of elasticity, and (3) test two key predictions of the wave‐speed flow limitation theory, namely (a) that the upstream pressure profile becomes independent of downstream pressure after flow limitation starts and (b) that the peak flowrate is given by (Dawson and Elliott 1977; Elliott and Dawson 1977)

(1) $$V_{I\max}=A\sqrt{\frac{A}{\rho C}}$$

where *A* is the cross‐sectional area of the choke point, *ρ = 1*.2 kg/m^3^ is the density of air, and $C=\frac{dA}{dP}$ is the tube compliance, where *P* is air pressure at the choke point. Our results shed some light on the physical mechanisms governing upper airway collapse in OSA.

## Methods

### Reconstruction of upper airway geometry

**Figure 2:**
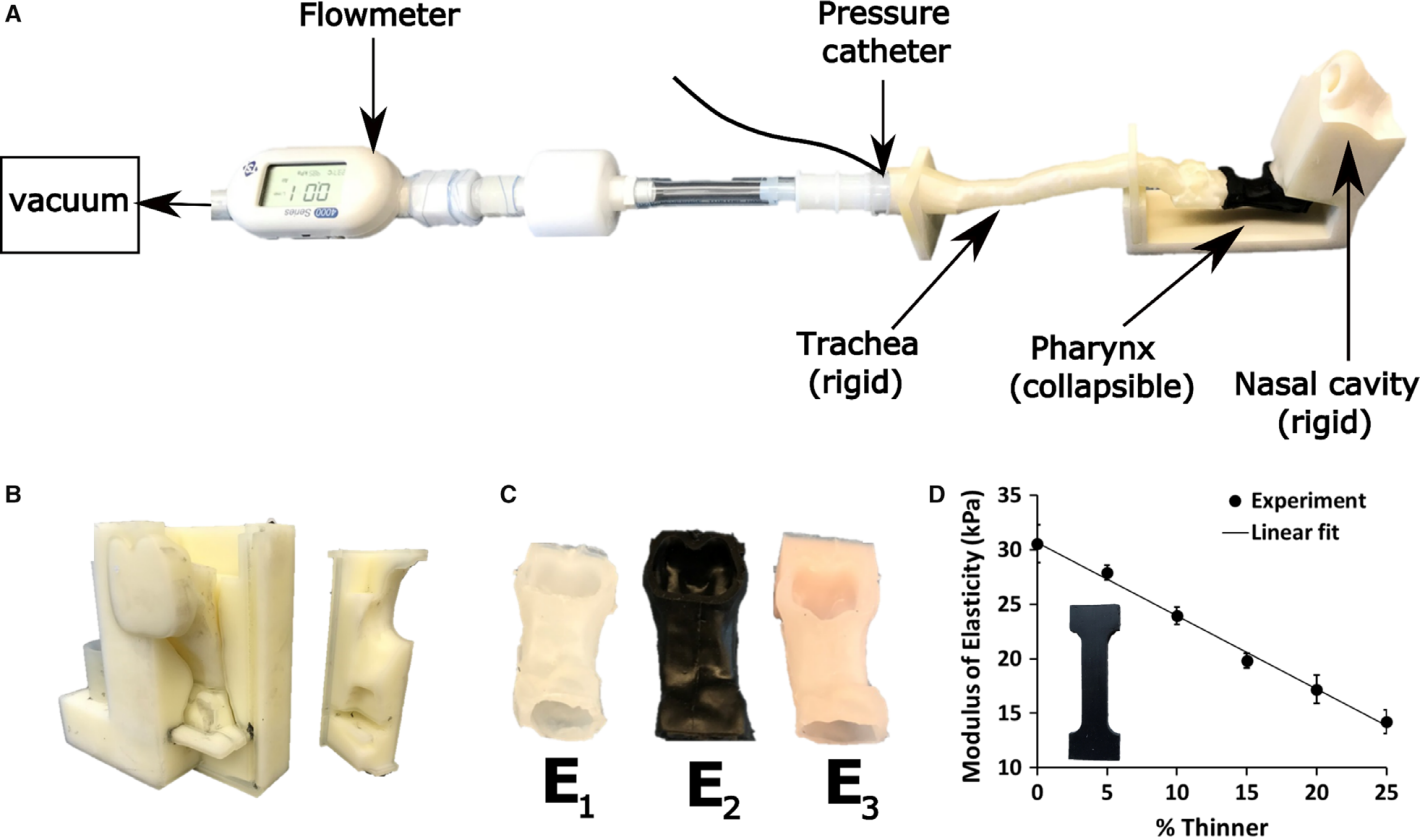
Experimental setup for measuring the pressure‐flow relationship in a replica of the human upper airway with a collapsible pharynx. (A) The anatomically accurate upper airway replica was created with a combination of 3D printing and silicone molding. Airflow was generated with laboratory vacuum. Downstream pressure was measured at the model outlet with a pressure catheter, while the flowrate was measured with a flowmeter. (B) 3D printed mold used to fabricate the collapsible pharynx. (C) Silicone pharynxes with varying modulus of elasticity. (D) Mechanical testing performed in dumbbell‐shaped testing samples (insert) revealed that the modulus of elasticity of the Ecoflex 00‐10 silicone decreased linearly with the amount of silicone thinner added to the mixture (percent of total weight).

**Figure 3:**
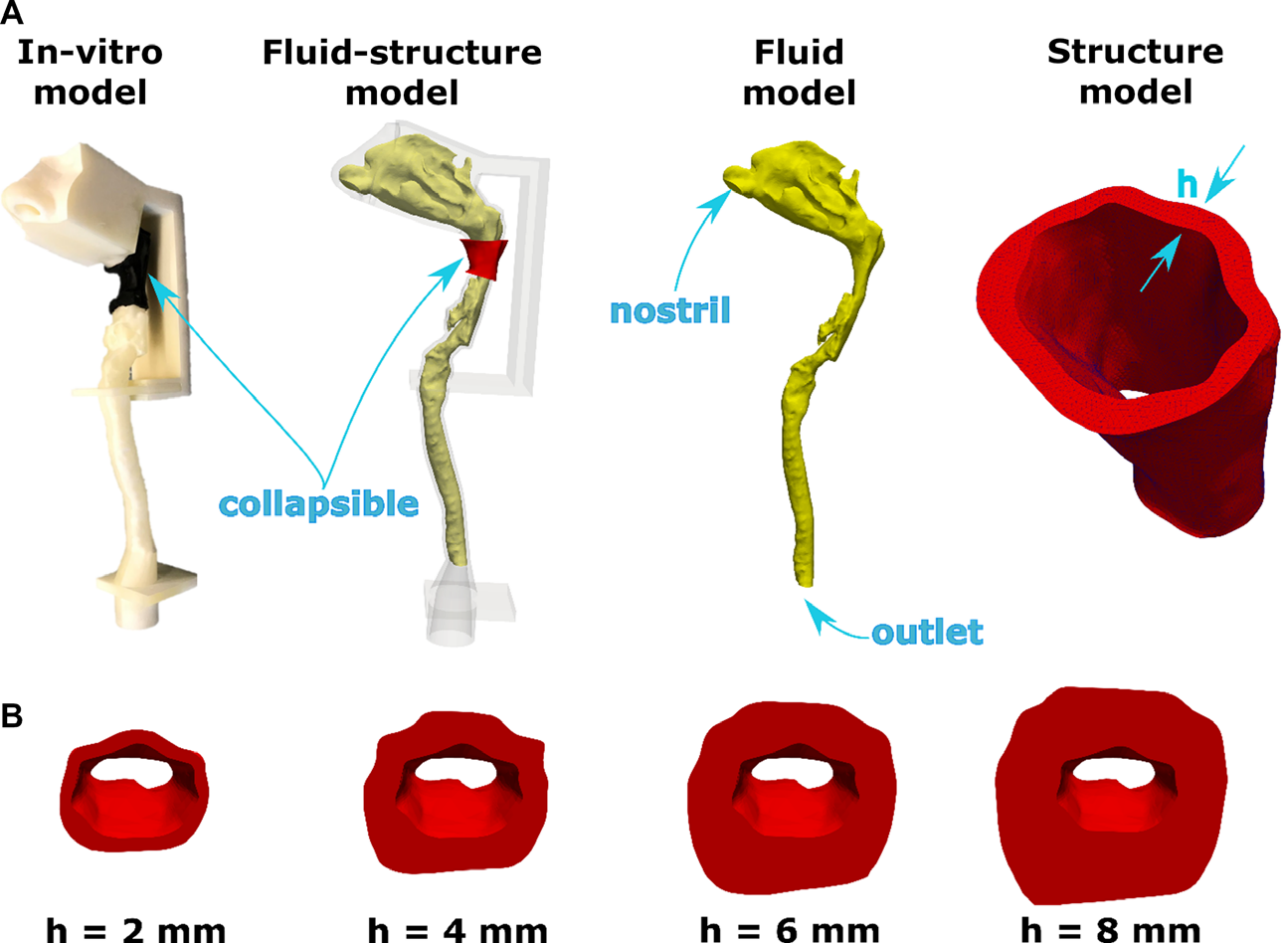
Fluid‐structure interaction model of the human upper airway. (A) The computational model was composed of a fluid domain and a structural domain. The fluid domain (yellow color) extended from nostrils to the end of the trachea. The structural domain (red color) was a segment of the pharynx with wall thickness h. (B) Structural models with wall thickness h = 2, 4, 6, and 8 mm.

Magnetic resonance images (MRI) of the head and neck of a healthy 49‐year‐old woman were obtained with approval from the Institutional Review Board at The Medical College of Wisconsin with informed consent signed by the volunteer. The selection of a healthy individual does not affect the general principles investigated in this study because upper airway collapse can be triggered in healthy individuals with subatmospheric nasal pressures (Schwartz et al. 1988). The three‐dimensional geometry of the upper airway was reconstructed in Mimics^™^ 19.0 (Materialise Inc., Leuven, Belgium). The upper airway model includes the geometry of the nasal cavity, pharynx, larynx, and trachea (Figs. 1, 2, 3). Inclusion of the nasal cavity is important to obtain realistic air pressures at the pharynx (Cisonni et al. 2015). The oral cavity was excluded, so that the model assumes a closed mouth.

The pharyngeal soft tissue was segmented with uniform wall thickness *h* around the airway perimeter (Fig. 3). Models with wall thickness varying from *h* = 2–8 mm were created to investigate the effect of wall thickness on airway compliance, while keeping the modulus of elasticity constant. Wall thickness was small in our models as compared to tissue thickness in OSA patients, but this was necessary to match the pharyngeal compliance of sleeping OSA patients using a silicone material that is stiffer than human pharyngeal tissues (see Discussion). The 3D model was exported from Mimics^™^ in STL format and imported into ANSYS SpaceClaim^™^ 18.1 (ANSYS Inc., Canonsburg, PA), where the surface mesh was smoothed and the model was prepared for 3D printing.

### Fabrication of collapsible airway replica

A physical replica of the upper airway (scale of 1:1) was fabricated using a combination of 3D printing and silicone molding (Fig. 2A). The rigid sections of the replica (nasal cavity, larynx, and trachea) were 3D printed using the 3D Systems Projet 3500 HD Max 3D printer, which has a resolution of 0.05 mm per 25.4 mm linear dimension. The printing material was the Visi‐jet M3‐X print media, which results in a hard plastic. The collapsible pharynx was fabricated using silicone molding. The mold was created with the same 3D printer and the same material used to print the rigid sections of the replica (Fig. 2B). The silicone Ecoflex 00‐10 (Smooth‐On Inc., Macungie, PA) was selected due to its low modulus of elasticity, tear resistance, compatibility with 3D printed parts, and the ability to vary the modulus of elasticity by adding a silicone thinner (Smooth‐on Inc.) (Gray et al. 2016). A vacuum chamber was used to remove all air bubbles from the silicone mixture prior to pouring the silicone into the mold. The silicone pharynx was allowed to cure for at least 48 h before being removed from the mold. The silicone pharynx has extended edges on both ends, so that it can be attached over the rigid ends of the replica. The unsupported, collapsible section of the pharynx has a length of 20 mm, which matches the FSI model.

The modulus of elasticity of the Ecoflex 00‐10 silicone was determined in the MTS Criterion^™^ Universal Testing System (MTS Systems Corporation, Eden Prairie, MN) using dumbbell‐shaped test samples and a tensile loading of 5 mm/sec. A linear relationship was observed between the modulus of elasticity (*E*) and amount of silicone thinner added to the mixture with modulus of elasticity decreasing from 30 kPa for 0% thinner to 15 kPa for 25% thinner by weight (Fig. 2D). This range is one order of magnitude larger than the modulus of elasticity reported for human pharyngeal tissue (*E* = 585–1409 Pa) (Birch and Srodon 2009). The Poisson's ratio of the Ecoflex 00‐10 silicone determined from the mechanical testing is 0.48 ± 0.02. The density of the Ecoflex 00‐10 silicone reported by the manufacturer is *ρ* = 1080 kg/m^3^.

### Experimental setup

The silicone pharynx was mounted on the rigid airway replica and the connections were sealed. The airway replica was placed horizontally on the bench as shown in Figure 2A. The nostrils were open to the atmosphere and a vacuum source was attached downstream of the model to generate airflow. A pressure catheter (Mikro‐Cath^™^, Millar Inc., Houston, TX) was used to measure pressure at the model outlet (end of the trachea), while a flowmeter (Model 4045, TSI Inc., Shoreview, MN) was used to measure the flowrate (Fig. 2A). These sensors were connected to a laptop via a USB hub and a data acquisition system.

**Figure 4:**
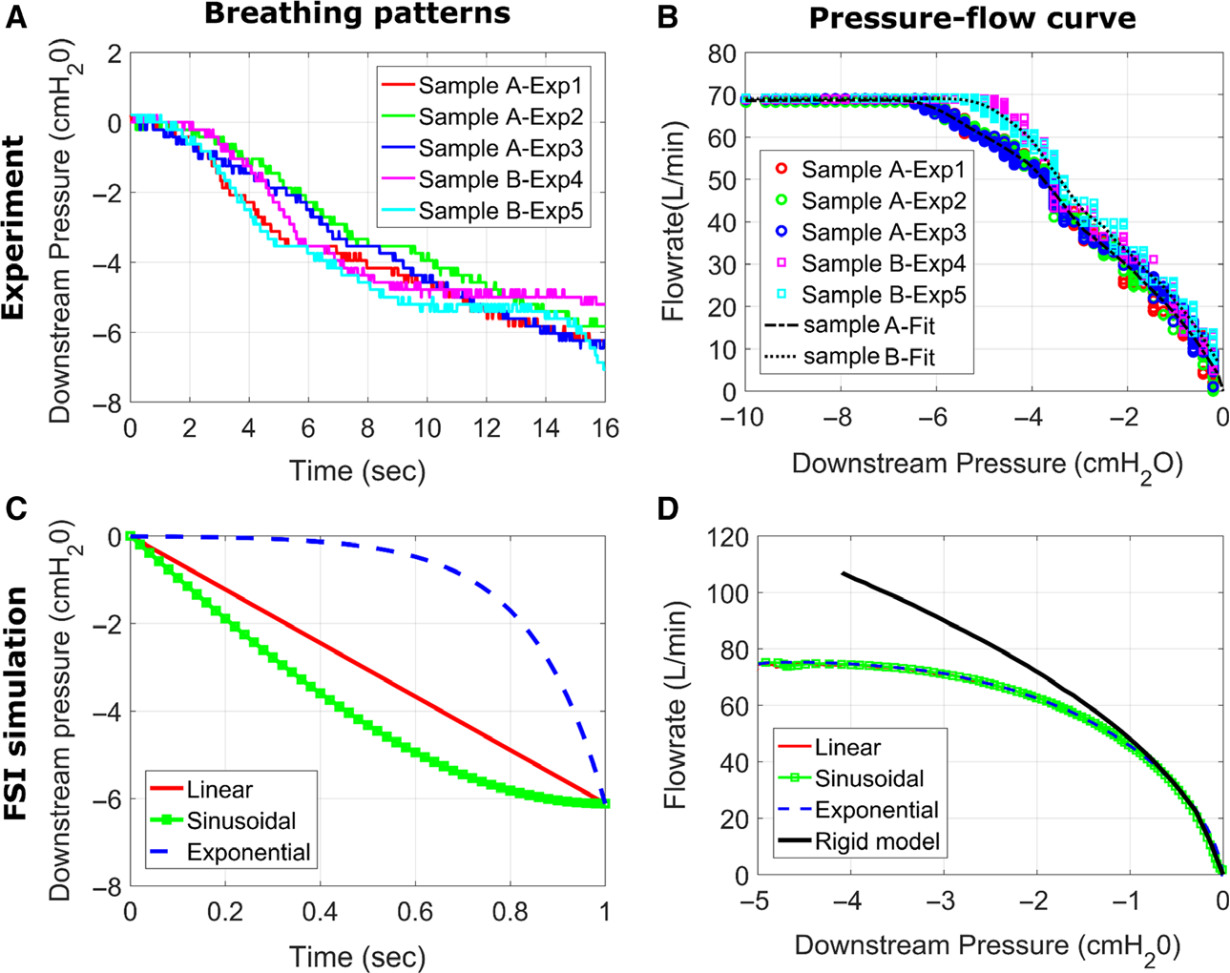
The pressure‐flow curve is independent of the breathing pattern. (A) Downstream pressure versus time in the five experiments performed in two replicas with modulus of elasticity E = 30 kPa. Variability between experimental runs was due to variation in the rate at which the vacuum valve was opened. (B) The pressure‐flow curve was nearly independent of the rate at which the vacuum valve was opened for each sample. (C) FSI simulations were performed for three different breathing profiles, namely linear, sinusoidal, and exponential profiles (see eqs. (3a), (3b), (3c)) in a model with wall thickness h = 8 mm and modulus of elasticity E = 2 kPa. (D) The pressure‐flow curve generated by the FSI simulations was independent of the breathing profile.

The experiment was performed by gradually opening the vacuum source valve until the silicone pharynx collapsed (Fig. 4A). The pressure drop across the healthy nasal cavity was insufficient to collapse the pharynx when both nostrils were open. To reduce air pressure at the pharynx, all experiments were performed by completely blocking the right nostril using tape. In other words, only the left nostril was open to the atmosphere in all experiments.

### FSI simulations

#### Numerical methods

Fluid‐structure interaction simulations were performed in ANSYS Workbench 18.1 (ANSYS Inc., Canonsburg, PA). The fluid domain was governed by the incompressible Navier‐Stokes equations and solved in ANSYS Fluent^™^ 18.1 using the standard air properties (air density *ρ = 1*.225 kg/m^3^ and air dynamic viscosity *μ = 1*.79 × 10^−5^ kg/m·sec). The structural domain was governed by Newton's second law and solved in ANSYS Mechanical^™^ 18.1. Data exchange between the fluid and structural fields was performed using ANSYS System Coupling^™^ 18.1.

The standard *k*‐*ω* model was adopted to account for turbulence in the airstream. To quantify turbulence, the *k*‐*ω* model solves two equations (in addition to the Reynolds‐averaged Navier‐Stokes equations), namely an equation for the turbulence kinetic energy (*k*) and an equation for the specific rate of dissipation (*ω*). This model was adopted for its low computational cost and accuracy, since previous studies have demonstrated that the standard *k*‐*ω* model accurately reproduces the pressure field in patient‐specific replicas of the human upper airway (Mylavarapu et al. 2009). Thus, the standard *k*‐*ω* turbulence model was used to simulate airflow with a turbulent scale of 1 mm and 5% turbulence intensity at the nostrils.

Silicone rubbers and biological tissues are hyperelastic materials and their nonlinear stress–strain relationship is well described by the Neo‐Hookean model (Subramaniam et al. 2016, 2017, 2018; Henrik Strand Moxness et al. 2018). This model describes the energy stored in the deformation via a strain energy density function, whose material constants *C*_1_ and D_1_ are related to the nominal modulus of elasticity *E* and Poisson's ratio *ν*:

(2a) $$\text{Neo‐Hookean coefficients}:C_{1}=\;\frac{\mu }{2}\;\;\text{and}\;\;D_{1}=\frac{\lambda }{2}$$

(2b) $$\begin{array}{c} \text{where the Lam}\overset{´}{e}\;\text{constants are}\;\lambda =\frac{\nu E}{\left(1+\nu )(1-2\nu \right)}\;\;\\ \text{and}\;\;\mu =\frac{E}{2(1+\nu )}. \end{array}$$

In ANSYS Fluent^™^, the second order upwind scheme was used to discretize the convective terms in the momentum equation, while the first order implicit scheme was used for temporal discretization. The coupled algorithm was used for the pressure‐velocity coupling. Pressure interpolation was done using the second order scheme. In ANSYS Mechanical^™^, the nonlinear solver with large deformation was set for the solid domain (hyperelastic material). The convergence criterion was set to 0.001 for globally scaled residuals of all flow variables. For each time step, five System Coupling^™^ iterations were performed. A time step of Δ*t* = 0.001 sec was sufficient to fulfill the convergence criteria. The FSI simulations were run on a workstation with 24 CPUs (Intel Xeon E5‐2687W v4 3.00 GHz) with 32 GB RAM memory. Each FSI simulation took 48–72 h wall‐clock time.

#### Boundary conditions

The boundary conditions for the fluid domain were set as follows: (a) no‐slip condition at all walls, (b) air pressure at both nostrils set to atmospheric pressure, and (c) air pressure at the outlet set to reproduce linear, exponential, or sinusoidal breathing profiles (see below). In a few cases (namely, in simulations aimed at investigating the effect of nasal resistance and in the comparison with experimental measurements), a wall boundary condition (i.e., zero flow) was applied to the right nostril to replicate the fact that the right nostril was blocked in the experiments. The boundary conditions for the structural domain were set as follows: (a) the inner wall of the structural domain was set as the FSI interface, which receives the loading condition from the flow field, (b) the external wall of the structural domain was set to atmospheric pressure (zero gauge pressure) and allowed to move freely, and (c) the anterior and posterior borders of the structural domain were fixed (i.e., zero displacement at the junctions between the flexible wall and rigid wall).

#### Mesh density

The fluid domain was discretized in ANSYS ICEM‐CFD^™^ 18.1, while the structural domain was discretized in ANSYS Mechanical^™^. A grid refinement study revealed that at least 300,000 tetrahedral cells in the fluid domain and a resolution of 1 mm for the solid domain were required for mesh‐independent results. Thus, a mesh with 355,000 tetrahedral cells in the fluid domain was adopted. The structural domain had 24,000 tetrahedral cells in the model with wall thickness *h* = 2 mm. These mesh sizes are similar to previous studies (Zhao et al. 2013; Pirnar et al. 2015). Dynamic mesh was enabled with boundary‐distance‐based diffusion smoothing and remeshing to maintain the quality of the deforming mesh. The smoothing algorithm was used to control the dynamic mesh and preserve the mesh near the moving boundary.

#### Initial condition

The initial condition for the FSI simulation (i.e., flow field at time *t* = 0 sec) was obtained by running a steady‐state simulation with rigid walls (zero displacement) and an outlet pressure of −1 Pa. This is necessary to avoid unphysical swings in air pressure in the initial System Coupling^™^ iterations that cause the software to crash when the simulations start from unrealistic pressure and velocity fields.

### Breathing profiles

To investigate how the breathing profile influences the dynamics of upper airway collapse, three different breathing profiles were studied (Fig. 4C):

(3a) $$\text{Linear profile}:p_{\mathrm{outlet}}=-600t$$

(3b) $$\text{Sinusoidal profile}:p_{\mathrm{outlet}}=-600\sin\left(\frac{\pi }{2}t\right)$$

(3c) $$\begin{array}{c} \text{Exponential profile}:p_{\mathrm{outlet}}=-600e^{k(1-t)}\;\;\\ \text{with}\;\;k=\ln\left(\frac{1}{600}\right) \end{array}$$

where *p*_outlet_ is the outlet pressure in Pa and *t* is time in seconds. The simulations were performed in the time domain *t* ϵ [0, 1] second. In this work, airway collapse was simulated only up to the point where opposite walls of the pharynx came into contact. In other words, contact between opposite walls was not simulated and the FSI simulation was stopped when the distance between opposite walls became smaller than 0.5 mm. The lowest pressure value of −600 Pa at the trachea was based on the experimental measurements.

### Definition of nasal resistance and airway compliance

Nasal resistance (R) was defined as the ratio of the transnasal pressure drop (*∆P*) to the flowrate (Q):

(4) $$R=\frac{\Delta P}{Q}$$

where *Q* is the sum of the flowrate in the left and right nostrils and *ΔP* = *P*_*nostrils*_ ‐ *P*_*pharynx*_ is the pressure drop from nostrils to pharynx. The assumption of atmospheric pressure at the nostrils imply that *P*_*nostrils*_ = 0 Pa (gauge pressure). Pharyngeal pressure (*P*_pharynx_) was defined as the area‐weighted average pressure across an axial section at the collapse site. In this work, nasal resistance was quantified at *∆P* = −15 Pa, so that *R* corresponds to the initial slope of the pressure‐flow curve at the collapse site.

Based on the wave‐speed flow limitation theory (Dawson and Elliott 1977; Elliott and Dawson 1977), airway compliance (*C*) is the slope of the area‐pressure curve (tube law) of the collapse site at the point where flowrate reaches its maximum value (i.e., $V_{I\max}$):

(5) $$C={\left(\frac{dA}{dP}\right)}_{V_{I\max}}$$

where A is the cross‐sectional area at the collapse site and *P* = *P*_pharynx_ is the area‐weighted average pressure at the collapse site.

## Results

### The pressure‐flow curve is independent of the breathing pattern

The impact of breathing profile (i.e., downstream pressure as a function of time) on the pressure‐flow curve of the upper airway was investigated experimentally in silicone pharynxes with wall thickness *h* = 2 mm (Fig. 4A and B). The vacuum valve was opened gradually and steadily over a period of approximately 16 sec. Since the valve was opened manually, the pressure versus time profile was different in each experimental run (Fig. 4A). However, different experimental runs provided the same pressure‐flow curve (Fig. 4B). The reproducibility of our experimental system was tested by fabricating two silicone pharynxes with Young's modulus *E* = 30 kPa. The pressure‐flow curves recorded in the two samples were only slightly different, and both samples had a peak inspiratory flow $V_{I\max}$ = 69 L/min (Fig. 4B).

The FSI simulations confirmed that the pressure‐flow curve is independent of the breathing profile for the values of wall thickness and modulus of elasticity studied. For example, FSI simulations were performed using linear, sinusoidal, and exponential breathing profiles (eqs. (3a), (3b), (3c)) in a model with wall thickness *h* = 8 mm and Young's modulus *E* = 2 kPa (Fig. 4C). All three breathing profiles provided the exact same downstream pressure versus flow curve (Fig. 4D). Therefore, all subsequent simulations in this study were performed using the linear breathing profile for simplicity.

### Validation of the computational model with in vitro experiments

**Figure 5:**
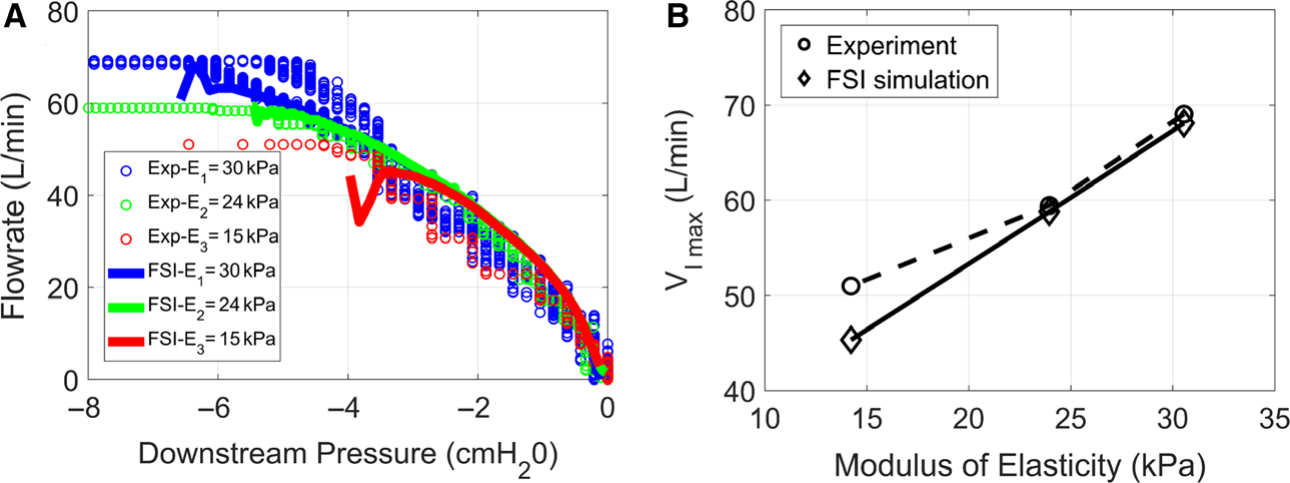
Experimental validation of FSI simulations. (A) Pressure‐flow curve measured experimentally and predicted by FSI simulations for moduli of elasticity E_1_ = 30 kPa, E_2_ = 24 kPa, and E_3_ = 15 kPa in a model with wall thickness h = 2 mm. (B) Maximum flowrate as a function of the modulus of elasticity.

The impact of the elastic properties of the pharyngeal wall on the pressure‐flow curve was investigated experimentally in silicone pharynxes with wall thickness *h* = 2 mm and modulus of elasticity *E* = 15, 24, and 30 kPa. The experiments revealed that the peak inspiratory flowrate increased as the modulus of elasticity increased (Fig. 5). FSI simulations in models with the same wall thickness and the same moduli of elasticity were in good agreement with the experiments. First, the initial slope of the pressure‐flow curve (where the collapsible pharynx behaves like a rigid structure, see Fig. 4D) was similar in the simulations and experiments (Fig. 5A). Second, the peak flowrate predicted by the FSI simulations was consistent with the experimental measurements (Fig. 5B). The good agreement between experimental measurements and FSI simulations despite the fact that the downstream pressure was dropped from 0 to −600 Pa in 16 sec in the experiments, but in only 1 sec in the simulations, further confirms that the pressure‐flow curve is independent of the breathing profile.

Some differences between the FSI simulations and experimental measurements must be noted. First, the FSI simulations did not reach downstream pressures as low as in the experiments (Fig. 5A). As opposite pharyngeal walls approached contact during airway collapse, the numerical simulations became unstable and stopped. This means that the FSI simulations represent the pressure‐flow curve before contact between opposite walls, while the experimental data include points both before and beyond wall contact. The sharp changes in flowrate near $V_{I\max}$ in some FSI simulations are a consequence of a tight coupling between airflow and wall displacement as the opposite walls approach contact (Fig. 5A). Note that these sharp changes in flowrate were not observed in the experiment. A second disagreement between the experimental measurements and FSI simulations is the large dispersion of the experimental data points for a given pressure value, especially at low pressures.

### The waterfall behavior: upstream pressure becomes independent of downstream pressure during flow limitation

**Figure 6:**
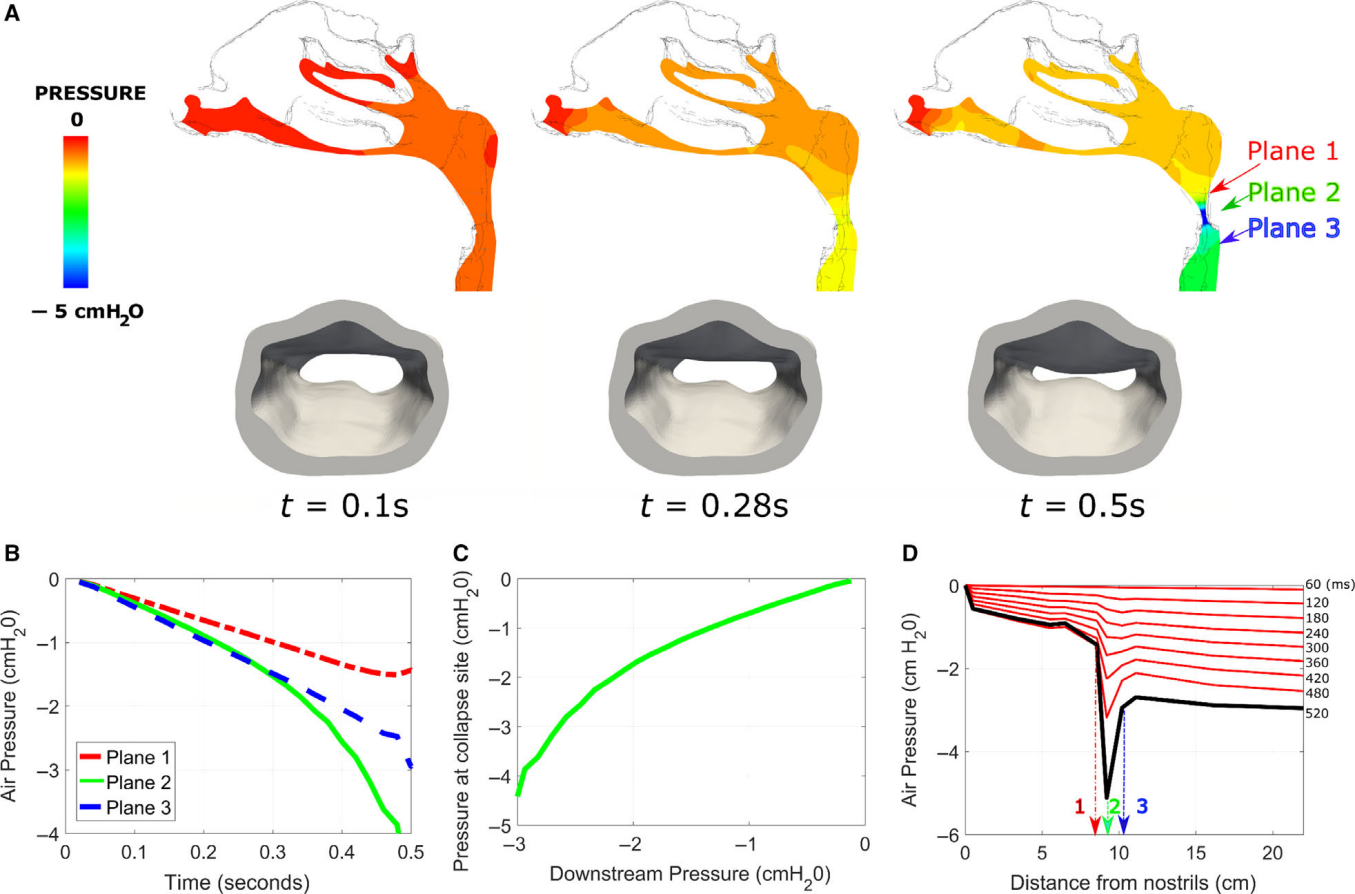
Air pressure at the collapse site is a nonlinear function of the downstream pressure. (A) Pressure field (top) and collapsible pharynx (bottom) at three time points during inspiration in a model with h = 2 mm, E = 15 kPa, and a linear breathing profile. Airway collapse occurred at plane 2. Planes 1 and 3 are located upstream and downstream of the collapse site respectively. (B, C) Air pressure at planes 1 and 3 decreased linearly with downstream pressure, but pressure at the collapse site was a nonlinear function of the downstream pressure. (D) Pressure versus distance from nostrils. Static pressure was averaged over planes perpendicular to the main flow direction. At flow limitation (thick black line), a local minimum in the pressure field is observed at the collapse site due to the Bernoulli effect. Downstream of the collapse site, pressure is approximately equal to the trachea pressure. Upstream of the collapse site, the pressure profile stops changing once the flow becomes limited (t > 400 msec).

One prediction of the wave‐speed flow limitation theory is that the pressure profile upstream of the choking point becomes independent of downstream pressure after flow limitation ensues (i.e., waterfall behavior). To test this prediction, FSI simulations were performed to investigate the time evolution of the pressure profile in a model with wall thickness *h* = 2 mm and modulus of elasticity *E* = 15 kPa (Fig. 6). Downstream pressure at the trachea was reduced linearly until opposite pharyngeal walls touched each other. As the pharynx collapsed, a choke point developed where airspace cross‐sectional area decreased sharply and air pressure developed a local minimum (Fig. 6A). To quantify the evolution of the pressure profile, air pressure was averaged over cross‐sections perpendicular to the main flow direction and plotted as a function of distance from nostrils in 60 msec intervals (Fig. 6D). Initially (*t* < 250 msec), air pressure at the pharynx was roughly halfway between its value at the nostrils and the tracheal outlet. As downstream pressure at the tracheal outlet continued to decrease and the pharynx collapsed, a sharp pressure gradient developed surrounding the choke point, so that a local minimum developed in the pressure profile (Fig. 6A and D). This behavior was due to a nonlinear relationship between pressure at the collapse site and downstream pressure (Fig. 6B and C). At flow limitation, pressure at the collapse site was substantially more negative than the outlet pressure, while downstream from the choke point the pressure profile was nearly flat (Fig. 6D). Importantly, the pressure profile upstream from the choke point became insensitive to changes in downstream pressure after flow limitation ensued (*t* > 400 msec), as predicted by the wave‐speed flow limitation theory.

### Effect of tissue elasticity on airway collapsibility

**Figure 7:**
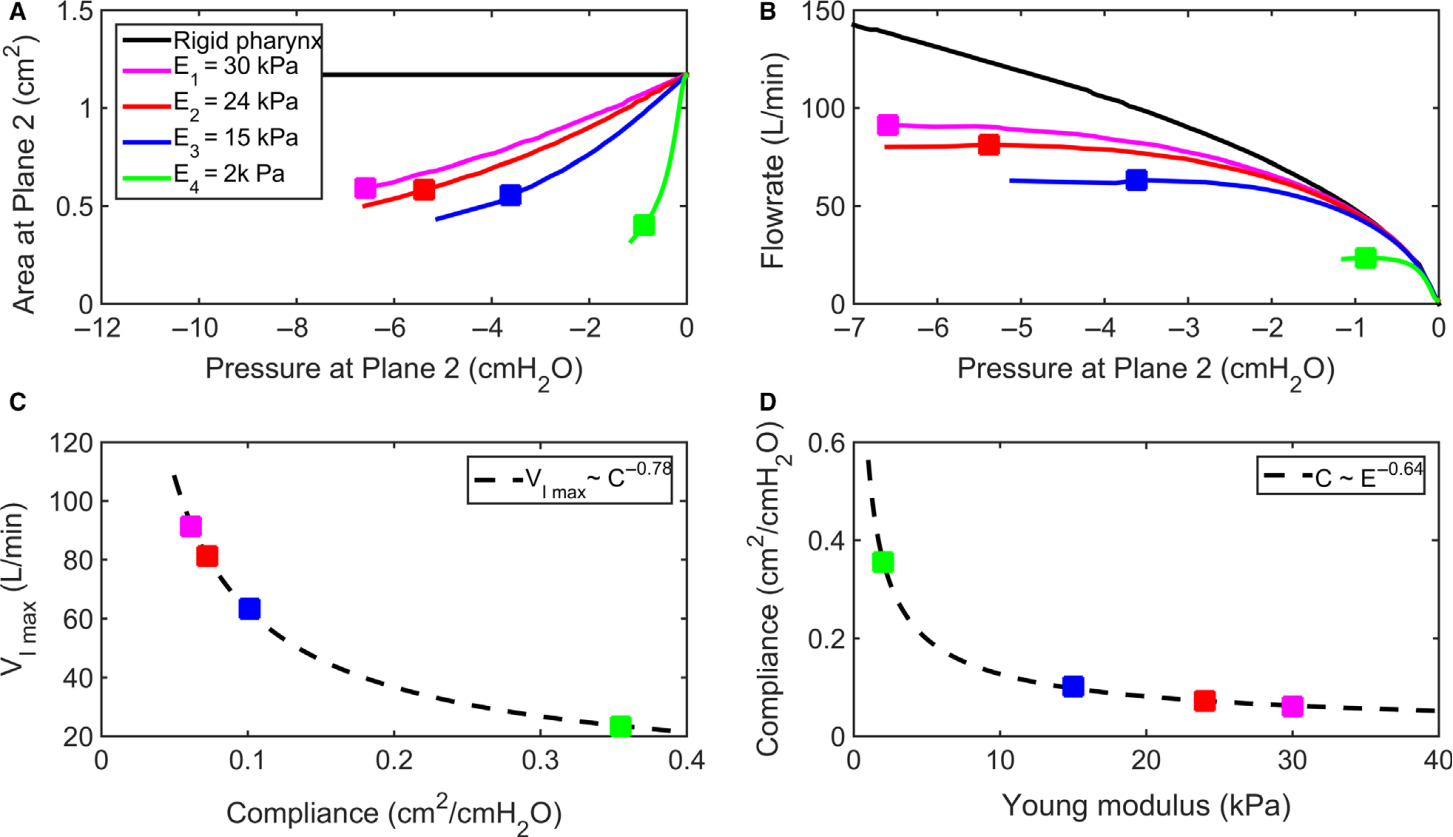
Effect of modulus of elasticity on airway collapsibility in a model with wall thickness *h* = 2 mm. (A) Area‐pressure relationship (tube law) at the collapse site (i.e., plane 2 in Fig. 6A) in models with modulus of elasticity *E* = 2, 15, 24 and 30 kPa. (B) The pressure‐flow curve reveals flow limitation in models with flexible walls as compared to a model with rigid walls. The square symbols show the location of the peak flow rate in each curve. (C) Peak flowrate ($V_{I\max}$) plotted against airway compliance ($C=\frac{dA}{dP}$ at $V_{I\max}$). (D) Airway compliance decreases as the modulus of elasticity increases.

To further investigate the effect of tissue elasticity on the mechanics of upper airway collapse, FSI simulations were performed in models with modulus of elasticity ranging from 2 to 30 kPa, while keeping the wall thickness constant (*h* = 2 mm) (Fig. 7). The pressure‐flow curve of models with a collapsible pharynx was identical to the behavior of a rigid pharynx for pressure magnitudes below the buckling pressure (Figs. 1C and 7B). Airflow limitation was observed in all models with the inspiratory flowrate reaching a plateau as downstream pressure decreased. (Square symbols mark the location of the peak inspiratory flowrate $V_{I\max}$ in Fig. 7.) The cross‐sectional area at the collapse site (plane 2 in Fig. 6) decreased from an initial value *A*_0_ = 1.18 cm^2^ to a minimum value around *A*_min_ = 0.5 cm^2^ for all models. This corresponds to a linear displacement of about 5 mm on each side of the pharyngeal wall. Pharyngeal compliance was defined as the slope of the area‐pressure curve at the collapse site and at $V_{I\max}$ (eq. (5)) (Fig. 7A). The model with the smallest modulus of elasticity (*E* = 2 kPa) had the highest compliance and thus the smallest $V_{I\max}$ (Fig. 7C). As the modulus of elasticity increased from 2 to 30 kPa, airway compliance decreased from 0.35 to 0.06 cm^2^/cmH_2_O and $V_{I\max}$ increased from 23 to 91 L/min (Figs. 7C and D).

### Effect of wall thickness on airway collapsibility

**Figure 8:**
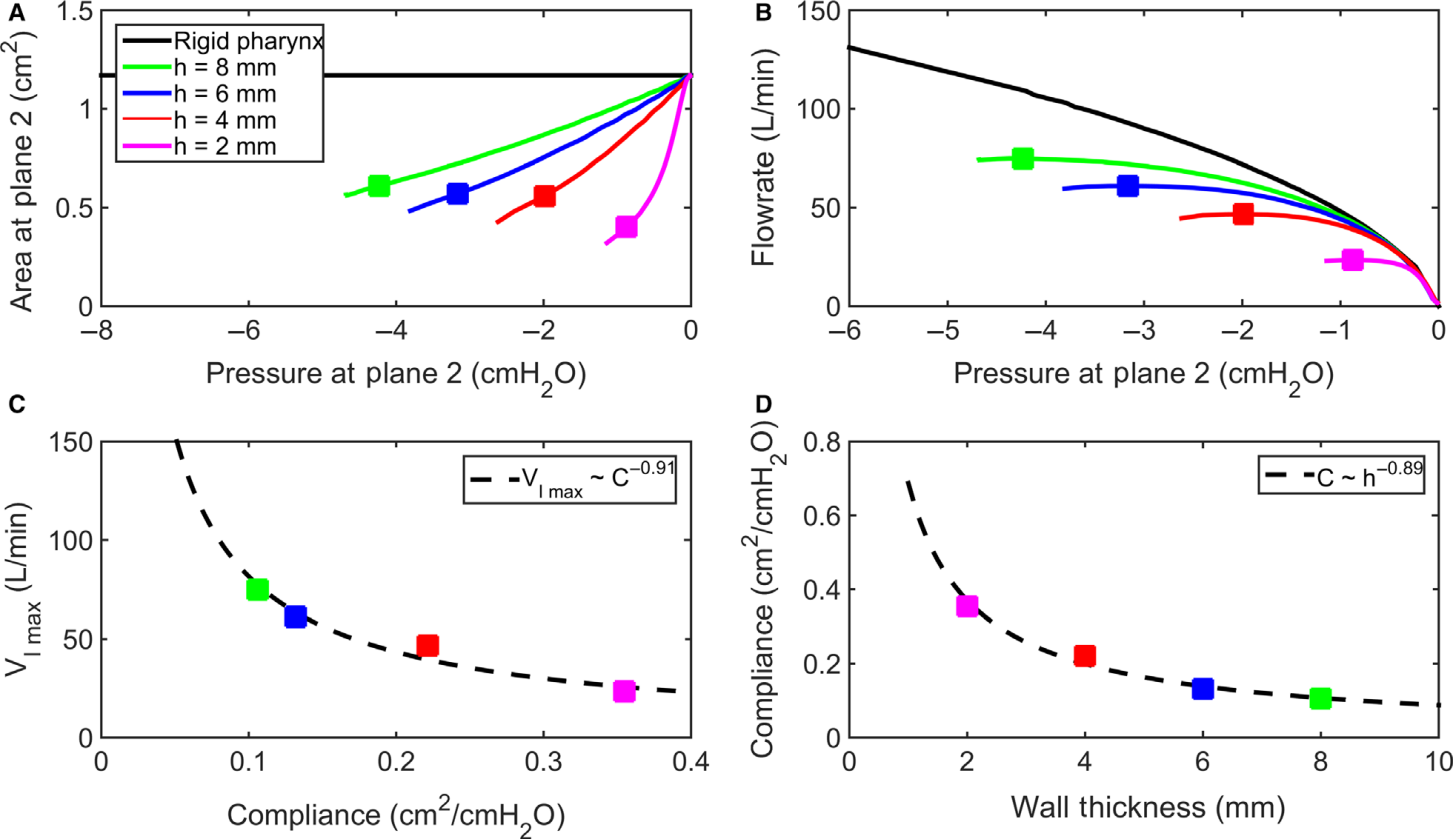
Effect of wall thickness on airway collapsibility in a model with modulus of elasticity *E* = 2 kPa. (A) Area‐pressure relationship (tube law) at the collapse site (i.e., plane 2 in Fig. 6A) in models with wall thicknesses 2, 4, 6, and 8 mm. (B) The pressure‐flow curve reveals flow limitation in models with flexible walls as compared to a model with rigid walls. The square symbols show the location of the peak flow rate in each curve. (C) Peak flowrate ($V_{I\max}$) plotted against airway compliance ($C=\frac{dA}{dP}$ at $V_{I\max}$). (D) Airway compliance decreases as the wall thickness increases.

FSI simulations were also performed to investigate the effect of wall thickness on the mechanics of upper airway collapse. FSI simulations were performed in models with wall thickness varying from *h* = 2–8 mm (Fig. 3), while assuming a constant modulus of elasticity *E* = 2 kPa. The FSI simulations revealed that wall thickness has a major impact on the tube law (Fig. 8A) and pressure‐flow curve (Fig. 8B) of the upper airway. The model with the smallest wall thickness (*h* = 2 mm) had the highest compliance and thus the smallest $V_{I\max}$. As wall thickness increased from 2 mm to 8 mm, airway compliance at the choke point decreased from 0.35 to 0.11 cm^2^/cmH_2_O (Fig. 8D) and $V_{I\max}$ increased from 23 to 75 L/min (Fig. 8C).

### Effect of nasal resistance on airway collapsibility

**Figure 9:**
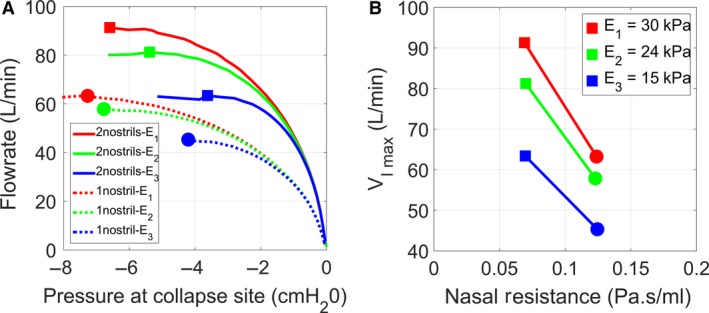
Effect of nasal resistance on airway collapsibility in a model with wall thickness *h* = 2 mm. (A) Pressure‐flow curve in models with modulus of elasticity *E*_1_ = 30 kPa, *E*_2_ = 24 kPa, and *E*_3_ = 15 kPa. The FSI simulations were performed either with both nostrils open to the atmosphere, or with the right nostril completely blocked to increase nasal resistance. (B) As the nasal resistance increases, the peak flowrate ($V_{I\max}$) decreases.

To investigate the effect of nasal resistance on airway collapsibility, FSI simulations were performed either with both nostrils open or with the right nostril blocked to simulate complete obstruction of the right cavity. Nasal resistance was defined as the initial slope of the pressure‐flow curve (eq. (4)). Blocking the right nostril increased nasal resistance from 0.07 to 0.12 Pa·sec/mL (Fig. 9). FSI simulations were performed for three values of the modulus of elasticity, namely *E* = 15, 24, or 30 kPa. The simulations demonstrated that nasal resistance has a major impact on the pressure‐flow curve of the upper airway (Fig. 9A) and that a reduction in nasal resistance leads to an increase in the peak flowrate that can be conducted by the collapsible upper airway (Fig. 9B). For example, in a model with *E* = 30 kPa, a reduction in nasal resistance from 0.07 to 0.12 Pa·sec/mL was associated with an increase in $V_{I\max}$ from 63 to 91 L/min.

### Comparison to the wave‐speed equation

**Figure 10:**
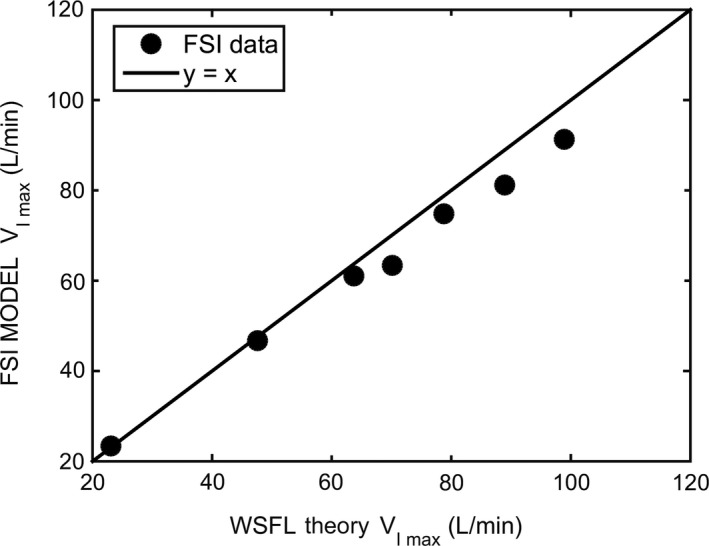
The peak flowrate ($V_{I\max}$) estimated by the FSI simulations is in good agreement with the value predicted by the wave‐speed flow limitation (WSFL) theory (eq. (1)).

To compare our FSI simulations to the WSFL theory, the choke point area and compliance at the instant when flow is maximum were inserted into equation (1). The $V_{I\max}$ predicted by the WSFL theory was in good agreement with the FSI simulations (Fig. 10). The agreement was better for models with high compliance (low $V_{I\max}$), but even in models with low compliance (high $V_{I\max}$) there was less than a 10% difference between the peak flowrate computed by FSI and predicted by the WSFL theory.

## Discussion

### The classical Starling Resistor model and OSA pathophysiology

The pathophysiology of OSA is complex. There is consensus that airway geometry and the mechanical properties of pharyngeal tissues play a major role in determining pharyngeal compliance (Dempsey et al. 2010). But neuromuscular factors, such as chemosensitivity, arousability, and sleep stage, also influence muscle tone and pharyngeal compliance (Dempsey et al. 2010, 2014; Carberry et al. 2016). In this work, we investigated how pharyngeal compliance is determined by tissue elasticity and wall thickness in a passive model of the human pharynx (i.e., muscle forces were not considered).

The classical Starling Resistor model explains many aspects of OSA pathophysiology (Smith et al. 1988; Gold and Schwartz 1996; Schwartz and Smith 2013). It correctly predicts that (1) the severity of airflow limitation during sleep is determined by the gradient between nasal pressure and tissue pressure, (2) $V_{I\max}$ increases linearly with nasal pressure, and (3) oscillations (snoring) can occur when tissue pressure exceeds lumen pressure, but remains below nasal pressure (Smith et al. 1988; Gold and Schwartz 1996; Schwartz and Smith 2013). However, the model's assumption that during flow limitation the flowrate is independent of downstream pressure was contradicted by the observation of negative effort dependence in OSA patients (Butler et al. 2013; Owens et al. 2014; Wellman et al. 2014; Genta et al. 2017) (Fig. 1C). This suggests that the classical Starling Resistor model is only an approximation of the biomechanics of upper airway collapse. It is also possible that the upper airway behaves like a classical Starling Resistor in some, but not all OSA patients. Several phenotypes of airway collapse have been described (Kezirian et al. 2011; Azarbarzin et al. 2017; Genta et al. 2017). Thus, it is unlikely that a single biomechanical model will describe airway collapse in all OSA patients (for example, epiglottic collapse is quite different from concentric collapse at the velopharynx).

### Wave‐speed flow limitation theory

The theoretical foundation behind the assumption that during flow limitation the flowrate becomes independent of downstream pressure is the WSFL theory. Flow limitation in collapsible tubes has been studied extensively (Bertram 2004, 2009) and the WSFL theory is supported experimentally (Elliott and Dawson 1977; Walsh et al. 1995; Bertram and Castles 1999) and theoretically (Shapiro 1977; Hyatt et al. 1981; Wilson et al. 2011; Pedersen and Butler 2011). However, one shortcoming of the WSFL theory is that, by itself, it does not identify the choke point and it does not provide the area‐pressure relationship (tube law) that is required to compute $V_{I\max}$ using equation (1). In fact, most theoretical analyses of the Starling Resistor are 1‐dimensional models based on a presumed tube law (Dawson and Elliott 1977; Shapiro 1977; Hyatt et al. 1981; Wilson et al. 2011; Pedersen and Butler 2011). Fluid‐structure interaction techniques provide a method to quantify the tube law (Figs. 7A and 8A) and the pressure‐flow curve (Figs. 7B and 8B) in 3‐dimensional, anatomically accurate models. Our FSI simulations are in good agreement with the WSFL theory, namely the pressure gradient upstream from the choke point became independent of downstream pressure once the flow became limited (Fig. 6D) and the peak flowrates in our models are in good agreement with the wave‐speed equation (Fig. 10). The time evolution of the pressure profile (Fig. 6D) in our FSI simulations is quite similar to in vivo measurements in sleeping OSA patients (Wellman et al. 2014), which suggests that our FSI models capture some important aspects of the dynamics of upper airway collapse. However, we did not observe negative effort dependence in our experiments or simulations, while NED is often observed in OSA patients (Butler et al. 2013; Owens et al. 2014; Wellman et al. 2014). Additional studies are needed to investigate the mechanical factors that regulate the emergence of NED. Butler and coauthors proposed that the profound negative effort dependence seen in some OSA patients cannot be explained by Starling resistors or wave speed theories, but rather requires a ‘lumped’ system in which a nonlocal tube law reflects the mechanical behavior of an entire segment (Butler et al. 2013).

### In vitro experiments

A key innovation of this study is the development of an experimental system to validate the FSI simulations. The modulus of elasticity of pharyngeal tissues is extremely low (*E* = 1 kPa) (Birch and Srodon 2009), thus creation of an anatomically accurate, collapsible model of the human pharynx is challenging. To our knowledge, only two previous studies reported in vitro experiments of human upper airway motion. Chouly and coauthors (2008) developed a simplified experimental model of the tongue using a water‐filled latex tube inserted perpendicular to a rigid pipe (Chouly et al. 2008). The simplified tongue was deformable, but still relatively stiff (*E* = 1600 kPa), thus airflow limitation was not studied. Zhao and colleagues (2013) used stereolithography to 3D print an anatomically accurate replica of the upper airway from an OSA patient. The replica was printed in hard plastic (*E* = 325 kPa), thus the wall deformation was relatively small and airflow limitation was not studied. In the present work, we identified a silicone material (Smooth‐On Ecoflex 00‐10) whose modulus of elasticity is only about 10 times higher than human pharyngeal tissue.

Although the modulus of elasticity of the Ecoflex 00‐10 silicone does not match the modulus of elasticity of human pharyngeal tissues, pharyngeal compliance in our experiments was near the range of pharyngeal compliance in sleeping OSA patients. Compliance is determined by both modulus of elasticity and geometry (wall thickness, curvature, etc.) (Figs. 7 and 8). To increase compliance, a small wall thickness (*h* = 2 mm) was employed in the experiments. We estimated that pharyngeal compliance in our in vitro model (*h* = 2 mm, *E* = 15 kPa; Fig. 7) was *C* = 0.10 cm^2^/cmH_2_O. This is near the range of pharyngeal compliances in sleeping obstructive sleep apnea patients (*C* = 0.28–1.76 cm^2^/cmH_2_O at the closing pressure) reported by Isono and coauthors (Isono et al. 1993). Importantly, the experiments in replicas with varying moduli of elasticity (*E* = 15 to 30 kPa) allowed us to validate our numerical methods (Fig. 5). Once validated, the FSI methods were applied to simulate more realistic combinations of modulus of elasticity and wall thickness, including a model with *E* = 2 kPa and *h* = 8 mm (Fig. 8). Pharyngeal compliance was estimated to be 0.35 cm^2^/cmH_2_O in this computational model, which is within the range of pharyngeal compliances reported by Isono and coauthors (1993). As predicted by the wave speed flow limitation theory, compliance is the key parameter that determines the peak flowrate (eq. (1)). Therefore, airway collapse in our models occurred in the physiological range of transmural pressures (0–10 cmH_2_O) and $V_{I\max}$ (Isono et al. 1993).

### Pharyngeal compliance is a function of airway geometry and tissue elasticity

Our results illustrate how the pressure‐flow profile reflects the geometric and elastic properties of the upper airway. The initial slope of the pressure‐flow curve represents the upstream resistance (Fig. 9A). As predicted by the classical Starling Resistor model (Fig. 1B), a reduction in nasal resistance increases the peak flowrate (Fig. 9B). For a constant nasal resistance, the peak flowrate is modulated by pharyngeal compliance. One strategy to reduce pharyngeal compliance is to increase the modulus of elasticity of soft tissues surrounding the airway (Fig. 7). This is illustrated by upper airway stimulation in OSA patients, where muscle stimulation makes the airway stiffer, thus preventing collapse (Strollo et al. 2014). In our model (based on a power law fitting to the FSI data), when modulus of elasticity increased twofold, pharyngeal compliance decreased 36%, and $V_{I\max}$ increased 41%.

Pharyngeal compliance is also modulated by airway geometry, including airspace cross‐sectional area, airway curvature, and soft tissue thickness (Dempsey et al. 2010; White and Younes 2012; Woodson 2015). In our model, a twofold increase in wall thickness reduced pharyngeal compliance by 46% and increased $V_{I\max}$ by 75%. This strengthening effect of increasing wall thickness while keeping the airspace cross‐sectional constant is well‐understood from a mechanical perspective (Kozlovsky et al. 2014), but it is more difficult to interpret in the context of OSA. Obese patients with OSA have thicker pharyngeal walls, but their pharyngeal compliance is higher (not lower) than normal (White and Younes 2012; Genta et al. 2014). This is explained by the fact that the upper airway is enclosed in a bony structure, so that an increase in wall thickness is coupled with a reduction in airspace cross‐sectional areas. Furthermore, obese patients may also have a higher tissue pressure (Gold and Schwartz 1996; Kirkness et al. 2008) (note however that the concept of tissue pressure remains controversial (Strohl et al. 2012)). Therefore, pharyngeal compliance is determined by a complex interplay between wall thickness, airspace cross‐sectional areas, and tissue pressure. Future studies are needed to investigate this complex relationship in 3D models that incorporate the bony enclosure that surrounds the upper airway.

### The pressure‐flow curve is independent of the breathing pattern

Our study suggests that the pressure‐flow curve is independent of the breathing pattern. FSI simulations performed with different time evolutions of the downstream pressure (i.e., sinusoidal, linear, or exponential profiles; Fig. 4C) provided the same pressure‐flow curve (Fig. 4D). Further evidence that the breathing pattern has a minimal impact on the pressure‐flow profile is the good agreement between our FSI simulations and in vitro experiments (Fig. 5) despite the fact that the pressure ramping was much slower in the experiment (as required for accurate flow measurements with our flowmeter). These findings are consistent with in vivo observations that the pressure‐flow curve in OSA patients preserves its shape over many breathing cycles (Wellman et al. 2014; Genta et al. 2017). The fact that the pressure‐flow profile is consistent and repeatable for each OSA patient is very important because it suggests that $V_{I\max}$ is a mechanical property of the airway and that it can be measured reliably in OSA patients. These findings suggest that in vivo measurements of $V_{I\max}$ during a sleep study can serve as a surrogate measure of pharyngeal compliance.

These observations suggest that flow limitation in the human upper airway may be approximated as quasi‐steady. The importance of time‐dependent effects in flow problems is governed by the Strouhal number St = *fL*/*V*, where f is a characteristic frequency, *L* is a characteristic length, and *V* is a characteristic velocity. When the characteristic frequency f is very small, so that *St *<< 1 , the flow is quasi‐steady, which means that it is well‐described by the steady Navier‐Stokes equations (Çengel and Cimbala 2006). In the absence of flow‐induced wall oscillations, the Strouhal nu mber is small (e.g., St = 0.001 for *L* = 1 cm = 0.01 m, *V* ≈ 2.5 m/sec, and $f=\frac{1}{4\;\text{sec}}$ = 0.25 Hz, where 4 sec is the period of the sinusoidal profile in our simulations), thus it is not surprising that the pressure‐flow curve was independent of the breathing pattern in our FSI simulations. However, wall oscillations (flutter) are often observed in Starling resistor models (Bertram and Tscherry 2006; Bertram 2008). The fact that we did not observe oscillations in our experiments or FSI simulations is consistent with previous experimental reports that flow limitation in collapsible tubes may occur with or without oscillations (Bertram and Castles 1999). Snoring is an important clinical manifestation of OSA. Future studies should investigate what mechanical factors regulate the emergence of oscillations (snoring) and its relationship to flow limitation.

### Study limitations and future directions

Several limitations of this work must be acknowledged. First, the pharynx had a nearly uniform wall thickness in our model. This approach allowed us to investigate systematically the effect of wall thickness on airway collapsibility. Future studies should account for the asymmetric distribution of tissue surrounding the pharynx and bony attachments that prevent soft tissue motion. Second, our FSI simulations were limited to airway collapse prior to contact of opposite walls. Future studies should develop numerical methods for modeling wall contact, which may be necessary to simulate negative effort dependence in OSA patients. Third, our study was limited to a single airway model that was based on an MRI from a healthy subject. Future studies that account for inter‐individual differences in upper airway anatomy may be able to demonstrate geometric and biomechanical factors that make OSA patients more susceptible to airway collapse. Finally, this study only investigated the passive behavior of the pharynx. Muscle activation is variable, which effectively means that airway elasticity is not constant. Future studies should investigate how the patterns of airway motion are affected by changes in muscle tone.

## Conclusions

In summary, we investigated the pressure‐flow curve in a collapsible model of the human upper airway using both in vitro and computational approaches. We examined the dependence of the shape of pressure‐flow curve on the geometric and mechanical properties of the pharyngeal wall and also on the breathing pattern. Our main findings are:

1. The peak flow in our FSI simulations was in good agreement with the wave speed equation;
2. The pressure profile upstream from the choke point became independent of downstream pressure after flow limitation ensued;
3. The pressure‐flow curve is independent of breathing pattern (i.e., downstream pressure vs. time profile);
4. The shape of the pressure‐flow curve provides significant information on the mechanical properties of the pharynx. In particular, the peak flowrate is inversely proportional to the square root of pharyngeal compliance ($V_{I\max}\propto C^{-1/2}$). This suggests that pharyngeal compliance in OSA patients may be estimated from peak flow measurements during sleep;
5. Pharyngeal compliance can be reduced by increasing the modulus of elasticity, but also by increasing the wall thickness;
6. The peak flowrate $V_{I\max}$ corresponds approximately to the instant when opposite walls of the pharynx come into contact;
7. A reduction in nasal resistance increases the peak flow $V_{I\max}$.

Future FSI studies are needed to confirm these findings and, in particular, to investigate airway collapse beyond the contact point.

## Conflict of Interest

The authors have no conflict of interest to disclose.
